# Motion correction in simultaneous PET/MR brain imaging using sparsely sampled MR navigators: a clinically feasible tool

**DOI:** 10.1186/s40658-015-0118-z

**Published:** 2015-07-16

**Authors:** Sune H Keller, Casper Hansen, Christian Hansen, Flemming L Andersen, Claes Ladefoged, Claus Svarer, Andreas Kjær, Liselotte Højgaard, Ian Law, Otto M Henriksen, Adam E Hansen

**Affiliations:** 1 3982 Department of Clinical Physiology, Nuclear Medicine and PET, Rigshospitalet, University of Copenhagen, Blegdamsvej 9, DK-2100 Copenhagen, Denmark; 2 6931 Neurobiology Research Unit, Rigshospitalet, University of Copenhagen, Blegdamsvej 9, DK-2100 Copenhagen, Denmark

**Keywords:** PET/MR, Motion correction, Motion quality control, Navigators, Rigid head motion, Clinical tools

## Abstract

**Background:**

We present a study performing motion correction (MC) of PET using MR navigators sampled between other protocolled MR sequences during simultaneous PET/MR brain scanning with the purpose of evaluating its clinical feasibility and the potential improvement of image quality.

**Findings:**

Twenty-nine human subjects had a 30-min [^11^C]-PiB PET scan with simultaneous MR including 3D navigators sampled at six time points, which were used to correct the PET image for rigid head motion. Five subjects with motion greater than 4 mm were reconstructed into six frames (one for each navigator) which were averaged to one image after MC.

The average maximum motion magnitude observed was 3.9 ± 2.4 mm (1 to 11 mm). Visual evaluation by a nuclear medicine physician of the five subjects’ motion corrected rated three of the five images blurred before motion correction, while no images were rated blurred after. The image quality was scored on a scale of 1–5, 5 being best. The score changed from an average of 3.4 before motion correction to 4.0 after. There was no correlation between maximum motion magnitude and rating. Quantitative SUVr scoring did not change markedly with motion correction.

**Conclusions:**

Sparsely sampled navigators can be used for characterization and correction of head motion. A slight, overall decrease in blurring and an increase in image quality with MC was found, but without impact on clinical interpretation. In future studies with noteworthy motion artifacts, our method is an important and simple-to-use tool to have available for motion correction.

**Electronic supplementary material:**

The online version of this article (doi:10.1186/s40658-015-0118-z) contains supplementary material, which is available to authorized users.

## Findings

### Introduction

Patient motion is a challenging problem in PET: it occurs regularly and is complex to estimate and correct for [8, 10–12]. The use of MR navigators for motion correction (MC) of PET in simultaneous PET/MR typically entails dense sampling of navigators during the full PET scan, preventing other MR sequences from being acquired [4, 18]. Alternatively, MR navigators can be acquired interleaved in other MR imaging, but this is currently only implemented for a very limited number of MR sequences [17]. These approaches with MR navigators, like many previous PET motion correction strategies, become too complex to be implemented in clinical routine and clinical research. We therefore present a study with an easily implemented MC setup using MR navigators sampled between other protocolled MR sequences during PET/MR brain scanning, to make motion quality control (QC) and motion correction clinically feasible. The purpose of this study is to evaluate the improvement in image quality and quantification with our method and assess its clinical feasibility.

### Material and methods

**Fig. 1:**
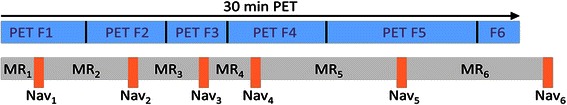
Setup of the PET/MR scan protocol used in this study. The PET framing (F1–F6) was set individually for each subject depending on the acquisition time of the navigators (Nav_1_–Nav_6_)

Twenty-nine human subjects (healthy controls and patients) in an ongoing ethically approved dementia study were included. During a 30-min [^11^C]-PiB (453 ± 148 MBq) PET scan in listmode on a Siemens Biograph mMR [5], 3D navigator volumes (2D EPI 3.0 × 3.0 × 3.0 mm^3^ voxels, 64 × 64 matrix, 36 slices, TE 30 ms, TR 3000 ms) were acquired in between other scheduled MR acquisitions at six time points (mean ± SD): 73 ± 10, 441 ± 13, 698 ± 62, 876 ± 129, 1412 ± 115, and 1947 ± 89 s after PET scan start. The scan setup is shown in Fig. 1. At each of the six sample points, 10 navigators (of 30 s duration in total) were acquired to check for noise in the navigator-to-navigator registration. The subjects were placed on the scanner bed to lie comfortably and their heads supported with foam wedges to restrict motion. The head coil does not allow room for a fixation band over the forehead.

Navigators 2–6 (Nav_2_–Nav_6_) were rigidly registered to the first navigator (Nav_1_) using SPM [16], and the motion magnitude for a point in the frontal cortex (60 mm in front of the scanners center of FOV) was calculated and plotted for assessment of maximum motion magnitude. This point was selected as the largest motion is typically seen in the front of the head [11]. The calculations of the motion magnitudes were done by applying the geometrical transformation matrix (in homogenous coordinates [7]) of a given navigator to the point (x, y, z, 1) = (0, 60, 0, 1) mm.

PET images were reconstructed with the mMR software employing OSEM-3D (4 iterations, 21 subsets, 5 mm FWHM Gaussian filter) using CT-based μ-maps [1].

**Fig. 2:**
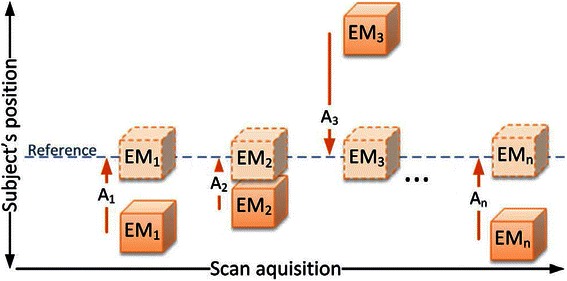
The principle of frame-by-frame motion correction to a common reference. In our study, the reference is the first PET frame (EM_1_) and thus there is no A_1_-transformation (courtesy of Oline Vinter Olesen, Technical University of Denmark)

The three subjects with the largest maximum motion magnitude (11, 8, and 8 mm) and the only two PiB-positive patients amongst the seven subjects with maximum motion magnitude >4 mm were selected for further analysis. Based on our prior work [11], MC will not have any (positive) effect at motion magnitudes >4 mm in this study. PET data was reconstructed into six frames, which were split at the midpoint between navigator start times (ensuring any PET data was linked to the nearest motion sample) and then averaged to one image after MC using the transformations obtained from the navigator registration. The framing was (mean ± SD) 259 ± 8, 306 ± 8, 191 ± 2, 342 ± 2, 546 ± 1, and 157 ± 19 s. The rigid motion correction of the PET frames was performed using the navigator positions and follows the principle of PET image-based motion correction, also known as multiple acquisition frames (MAF) [14] as shown in Fig. 2. As with PET image-based MC, we assume no or negligible intra-frame motion.

A blinded evaluation comparing non-MC and MC PiB images was performed by a nuclear medicine physician specialized in neuroimaging doing both qualitative reading and quantitative analysis, the latter as SUVr (cerebellum to region ratio of SUVs (standardized uptake value)) calculated in the Siemens Syngo.via Scenium ratio analysis tool using a set of cortical standard regions [6]. SUVr >1.5 was considered an abnormal PiB uptake.

### Results

**Fig. 3:**
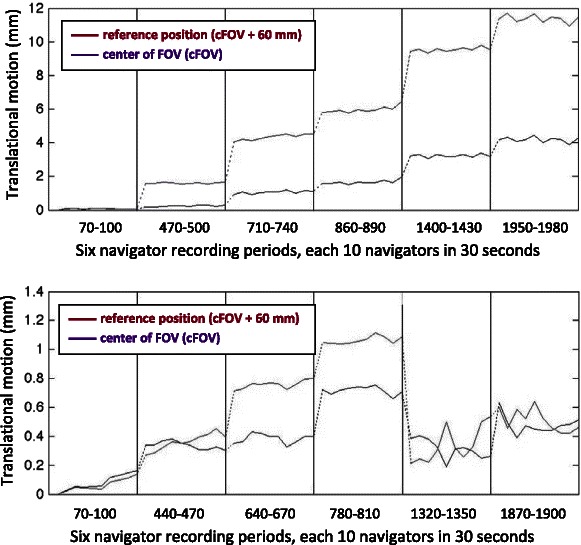
Plots of measured motion magnitude. Fifty-nine navigator volumes were registered to the first navigator (Nav_1,1_, reference position). The subject with the largest maximum motion magnitude is shown at the *top* and the subject with the lowest maximum motion magnitude (1 mm) at the *bottom*. The *red curves* are the motion at the 60 mm point representing cortex motion and is the reference point for motion magnitude in our study. The *purple curves* are the motion magnitude at the scanners center of FOV (cFOV). The individual translational and rotational components of the motion are given in the Additional file 1 (part 2)

The average of the maximum motion magnitudes measured over the 29 scans, each 30 min long, was 3.9 ± 2.4 mm (range: 1 to 11 mm). The motion for two subjects with maximum and minimum magnitudes are plotted in Fig. 3 and show very low noise/internal motion between the 10 navigators (Nav_n,1_–Nav_n,10_) in each of the 30-s sampling periods (less than 1 mm in all 29 subjects).

**Fig. 4:**
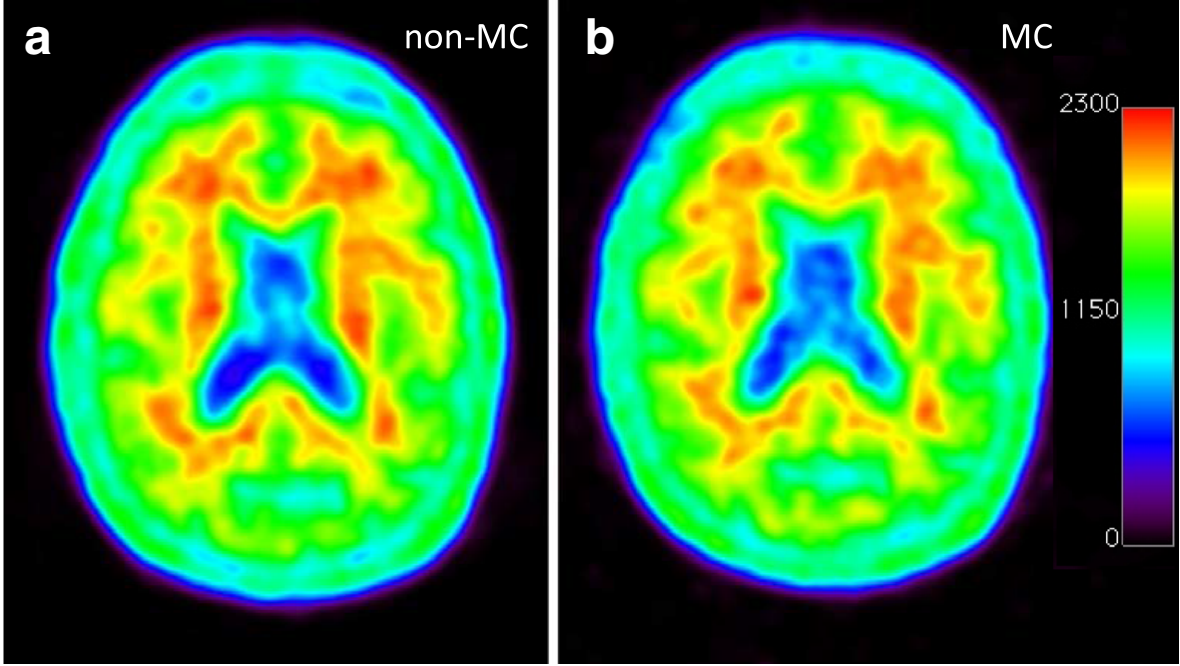
Visual results of motion correction. Subject five with an 11-mm maximum motion magnitude before MC in (**a**) and after MC in (**b**). The visual improvement with motion correction is minor, and our PiB images are as such not highly sensitive to motion artifacts (unless motion is unusual severe)

Results of both the visual and quantitative evaluation of the five motion corrected subjects are given in Table 1. Before motion correction, three of the five subjects were rated slightly blurred (8, 6, and 8 mm maximum motion magnitude, respectively) with no correlation between motion magnitude and rating. After motion correction, none of the images were rated as blurred. However, the improvement in image quality did not have clinical impact, as it was also observed in the quantitative scoring, where no noteworthy changes in SUVr following motion correction were seen. An example of the visual effect of motion correction is shown in Fig. 4.

**Table 1:** Visual (qualitative) and quantitative scoring of the five motion-corrected subjects before and after MC

Subject	PiB diagnosis	Motion at cFOV + 60 mm (frontal cortex)	Qualitative scoring				Quantitative scoring SUVr (PiB neg. if <1.5)	
			Blurred?		Rating: 1–5(best)			
			Non-MC	MC	Non-MC	MC	Non-MC	MC
S1	Negative	8 mm	No	No	4	4	1.06	1.07
S2	Positive	8 mm	Yes	No	3	4	2.59	2.67
S3	Positive	6 mm	Yes	No	3	4	3.01	3.05
S4	Negative	8 mm	Yes	No	3	4	1.05	1.04
S5	Negative	11 mm	No	No	4	4	1.08	1.08
Mean ± SD					3.4 ± 0.5	4.0 ± 0.0

### Discussion

Sparsely sampled MR navigators can be used for characterization, check for (QC), and correction of head motion in simultaneous PET/MR. A slight, overall decrease in blurring and an increase in image quality rating with MC was found, but with no impact on clinical interpretation. Decreased blurring was seen in the two patients with a PiB-positive scan and in one of the PiB-negative subjects (Table 1) but not in the two other PiB-negative subjects with the same or larger maximum motion magnitude, even though one would expect motion blurring (and the effect of correcting for it) to be proportional to motion magnitude. We note that motion magnitude alone does not account for all motion details, the magnitudes in question are close to each other, and our blurring score is subjective with only minor changes reported. The missing correlation between motion magnitude and image blurring might also be caused by the image resolution (scanner and tracer) of >5 mm being relatively high compared to the motion magnitudes ≤11 mm. Quantitative SUVr scoring does not change with MC, showing that scoring using cortical standard regions is robust against (minor/medium) motion, likely due to the use of large regions and low image contrast around them.

Our setup is simple and can be implemented with little effort on PET/MR scanners with simultaneous acquisition, as the MR navigator used here is standardly available. The duration of the MR sequences in the protocol used here (3–6 min) are typical for diagnostic MR of the brain and matches well with suitable PET frame duration for motion correction [11]. Here, densely sampled navigators [4, 18] would block MR time, and using integrated/interleaved navigators [17] for MC would severely limit the number of MR sequences available.

Sparsely sampled navigators as well as PET image-based MC [11] yield motion correction at a lower, but clinically sufficient, time rate than densely sampled navigators would do. PET image-based MC is tracer dependent, possibly resulting in lower spatial accuracy of the motion estimates. Sparsely sampled navigators do not suffer from this dependency, but their time framing depends on sampling availability, as determined by the MR protocol used and will still suffer from remaining intra-frame motion like PET image-based MC does.

The noise properties as a function of frame length are investigated in Additional file 1 (part 1) of this paper. The 3–6 min frame lengths used here for MC does not seriously compromise the signal to noise ratio (SNR) [9] as compared to the standard 30-min reconstruction, and motion correction at very high frequencies would require line of response-based PET reconstruction methods [2, 3, 15]. That the frame length is sufficient and does not miss major intra-frame motion (except for extraordinary cases of erratic motion) is in concurrence with our previous work [13]. Thus, our method would be useful for rough correction of larger patient motions, but will not do high level motion correction, e.g., in Parkinson’s patients.

We recorded 10 navigators in each sampling set requiring a 30-s recording time, but the low noise internally in each sampling set (Fig. 3) shows that one navigator would be enough per sampling in future use of this method, making it very time efficient and clinically feasible.

The dominant orientations of head motion in brain imaging [11] are from patients nodding their head and sliding downwards out of the scanner, which we also observed in this study on the mMR.

Although the effects of MC is limited in this PiB study, effects in longer (dynamic) scans, scans with larger motion magnitudes, other tracers (e.g., FDG) or smaller ROIs are expected, and the proposed navigator setup can easily be implemented for motion QC and MC in clinical practice with minimal adaption of standard scan protocols: on recent 40 min. [^18^ F]-FET pediatric studies we have applied sparsely sample navigator MC to aid accurate (quantitative) diagnostics. A similar system with PET image-based motion QC and MC [11] has proven useful at our HRRT brain scanner where we have seen MC with 5 min framing greatly improving the image quality.

### Conclusion

Sparsely sampled MR navigators can be used for motion QC and correction of head motion in simultaneous PET/MR. Image quality is slightly improved and blur is decreased but with no impact on clinical interpretation in the present study with limited maximum patient motion magnitude (1–11 mm). Our method has limitations compared to advanced MC methods with denser motion sampling, but in case of noteworthy artifacts from larger patient head motion, we have described a simple-to-use tool, easily implemented for motion correction in clinical practice.

## Additional file

Additional file 1:
**Part 1: dependency of image noise properties on frame length. Part 2: individual motion components for the two example patients in Fig.** 3 **.**

## References

[1] Andersen FL, Ladefoged CN, Beyer T, Keller SH, Hansen AE, Højgaard L (2014). Combined PET/MR imaging in neurology: MR-based attenuation correction implies a strong spatial bias when ignoring bone. Neuroimage.

[2] Bloomfield PM, Spinks TJ, Reed J, Schnorr L, Westrip AM, Livieratos L (2003). The design and implementation of a motion correction scheme for neurological PET. Phys Med Biol.

[3] Carson RE, Barker WC, Liow J-S, Johnson CA (2003). Design of a motion-compensation OSEM list-mode algorithm for resolution-recovery reconstruction for the HRRT. IEEE Nucl Sci Symp Conf Rec.

[4] Catana C, Benner T, van der Kouwe A, Byars L, Hamm M, Chonde DB (2011). MRI-assisted PET motion correction for neurologic studies in an integrated MR-PET scanner. J Nucl Med.

[5] Delso G, Fürst S, Jakoby B, Ladebeck R, Ganter C, Nekolla SG (2011). Performance measurements of the Siemens mMR integrated whole-body PET/MR scanner. J Nucl Med.

[6] Fleisher AS, Chen K, Liu X, Roontiva A, Thiyyagura P, Ayutyanont N (2011). Using positron emission tomography and florbetapir F 18 to image cortical amyloid in patients with mild cognitive impairment or dementia due to Alzheimer disease. Arch Neurol.

[7] Foley JD, van Dam A, Feiner SK, Hughes JF (1996). Computer graphics: principles and practice in C.

[8] Fürst S, Grimm R, Hong I, Souvatzoglou M, Casey ME, Schwaiger M (2015). Motion correction strategies for integrated PET/MR. J Nucl Med.

[9] Jakoby BW, Bercier Y, Conti M, Casey ME, Bendriem B, Townsend DW (2011). Physical and clinical performance of the mCT time-of-flight PET/CT scanner. Phys Med Biol.

[10] Keller SH, Hansen AE, Holm S, Beyer T, Carrio I, Ros P (2014). Image distortions in clinical PET/MR imaging. PET/MRI.

[11] Keller SH, Sibomana M, Olesen OV, Svarer C, Holm S, Andersen FL (2012). Methods for motion correction evaluation using FDG human brain scans on a high resolution PET scanner. J Nucl Med.

[12] Klausen TL, Keller SH, Olesen OV, Aznar M, Andersen FL (2012). Innovations in PET/CT. Q J Nucl Med Mol Imaging.

[13] Olesen OV, Keller SH, Sibomana M, Larsen R, Roed B, Højgaard L (2010). Automatic thresholding for frame-repositioning using external tracking in PET brain imaging.

[14] Picard Y, Thompson CJ (1997). Motion correction of PET images using multiple acquisition frames. IEEE Trans Med Imaging.

[15] Rahmim A, Dinelle K, Cheng JC, Shilov MA, Segars WP, Lidstone SC (2008). Accurate event-driven motion compensation in high-resolution PET incorporating scattered and random events. IEEE Trans Med Imaging.

[16] SPM Documentation (2014). Wellcome Trust Centre for Neuroimaging.

[17] Tisdall MD, Hess AT, Reuter M, Meintjes EM, Fischl B, van der Kouwe AJ (2012). Volumetric navigators for prospective motion correction and selective reacquisition in neuroanatomical MRI. Magn Reson Med.

[18] Ullisch MG, Scheins JJ, Weirich C, Rota Kops E, Celik A, Tellmann L, et al. MR-based PET motion correction procedure for simultaneous MR-PET neuroimaging of human brain. PLoS One. 2012;7. doi:10.1371/journal.pone.0048149 10.1371/journal.pone.0048149 PMC3495949 23189127

