# Erratum to: Motion correction in simultaneous PET/MR brain imaging using sparsely sampled MR navigators: a clinically feasible tool

**DOI:** 10.1186/s40658-015-0126-z

**Published:** 2015-10-06

**Authors:** Sune H. Keller, Casper Hansen, Christian Hansen, Flemming L. Andersen, Claes Ladefoged, Claus Svarer, Andreas Kjær, Liselotte Højgaard, Ian Law, Otto M. Henriksen, Adam E. Hansen

**Affiliations:** 3982 Department of Clinical Physiology, Nuclear Medicine and PET, Rigshospitalet, University of Copenhagen, Blegdamsvej 9, Copenhagen, DK-2100 Denmark; 6931 Neurobiology Research Unit, Rigshospitalet, University of Copenhagen, Blegdamsvej 9, Copenhagen, DK-2100 Denmark

## Erratum

Unfortunately, the original version of this article [1] contained an error. The additional file contained citation errors. The link to the correct additional file can be found below.

## Additional file

Additional file 1:
**Part 1: dependency of image noise properties on frame length.** Part 2: individual motion components for the two example patients in Fig. 3.
