# Supplementary material for: Motion correction in simultaneous PET/MR brain imaging using sparsely sampled MR navigators: a clinically feasible tool
Source: EJNMMI Phys. 2015 Jul 16;2:14. doi: 10.1186/s40658-015-0118-z (PMC4538713; doi:10.1186/s40658-015-0118-z)
Supplement: Additional file 1: — Part 1: dependency of image noise properties on frame length. Part 2: individual motion components for the two example patients in Fig. 3 . [file 40658_2015_118_MOESM1_ESM.pdf]

Supplementary material for:

Motion correction in simultaneous PET/MR brain imaging using sparsely sampled MR navigators: a clinically feasible tool

Sune H. Keller[1], Casper Hansen[1], Christian Hansen[1], Flemming L. Andersen[1], Claes Ladefoged[1], Claus Svarer[2], Andreas Kjær[1], Liselotte Højgaard[1], Ian Law[1], Otto M. Henriksen[1], Adam E. Hansen[1]
[1]*Dept. of Clinical Physiology, Nuclear Medicine and PET, Rigshospitalet (University of Copenhagen)*
[2]*Neurobiology Research Unit, Rigshospitalet (University of Copenhagen)*

Part 1: Dependency of image noise properties on frame length

To investigate the noise properties when using an average of a number of short frames as it is typically done in motion correction [14], we reconstructed the 30 minute scans of 3 subjects with minor motion (≤ 4 mm) in 1, 3 and 5 minute frames, combined them to 30 minute images by averaging and calculated their signal-to-noise ratios (SNR) as in [9]

$$SNR = (\mu_{signal} - \mu_{background})/\sigma_{background}. \quad (1)$$

μ_{signal} is the (mean) activity in a VOI masking the brain (volumes: 2177; 2142 or 2306 cm^3), $\mu_{background}$ is the activity and $\sigma_{background}$ is the noise (as standard deviation) in a background VOI in the neck with low PiB uptake (volumes: 34.1; 33.0 and 43.0 cm^3). Using subjects with minor motion excludes effects from motion itself or from transformations/resampling if MC is performed. (No motion correction was done in this substudy on noise.)

Results: Investigating the noise properties of using an average of short times frames, we found that the SNR increases with the length of the time frames for all 3 subjects included in this test as reported in **Table S1**. We also saw that the SNR of the standard 30 minute reconstruction fell between the SNRs of averaged 3 and 5 minute frames for all 3 subjects.

Discussion: The evaluation of SNR depending on frame length in **Table S1** shows that some improvement in SNR comes from the inherent smoothing in averaging the frames and an average of 5 minute frames has higher SNR than a 30 minute frame for all 3 subjects tested. This is most likely because of the smoothing from adding the frames, which improves SNR (a little) and that 5 minute of PET data is enough to have (close to) optimal signal to noise properties for reconstruction.
Thus the 3-6 minutes frame length used here for MC does not seriously compromise SNR as compared to the standard 30 minute reconstruction, but motion correction at very high frequencies would require line of response-based PET reconstruction methods [2–3, 15].

Table S1. **Signal-to-noise ratios for 30 minute images of 3 subjects with minor motion.** The regular 1-frame reconstruction is compared to average images composed of 1, 3 or 5 minute frames.

	SNR of the averaged images			
**Subject**	**30 x 1 min**	**10 x 3 min**	**6 x 5 min**	**1 x 30 min**
**S1**	1.65	1.69	1.73	1.70
**S2**	2.94	3.27	3.45	3.31
**S3**	1.79	1.88	1.98	1.97

Part 2: Individual motion components for the two example patients in Figure 3

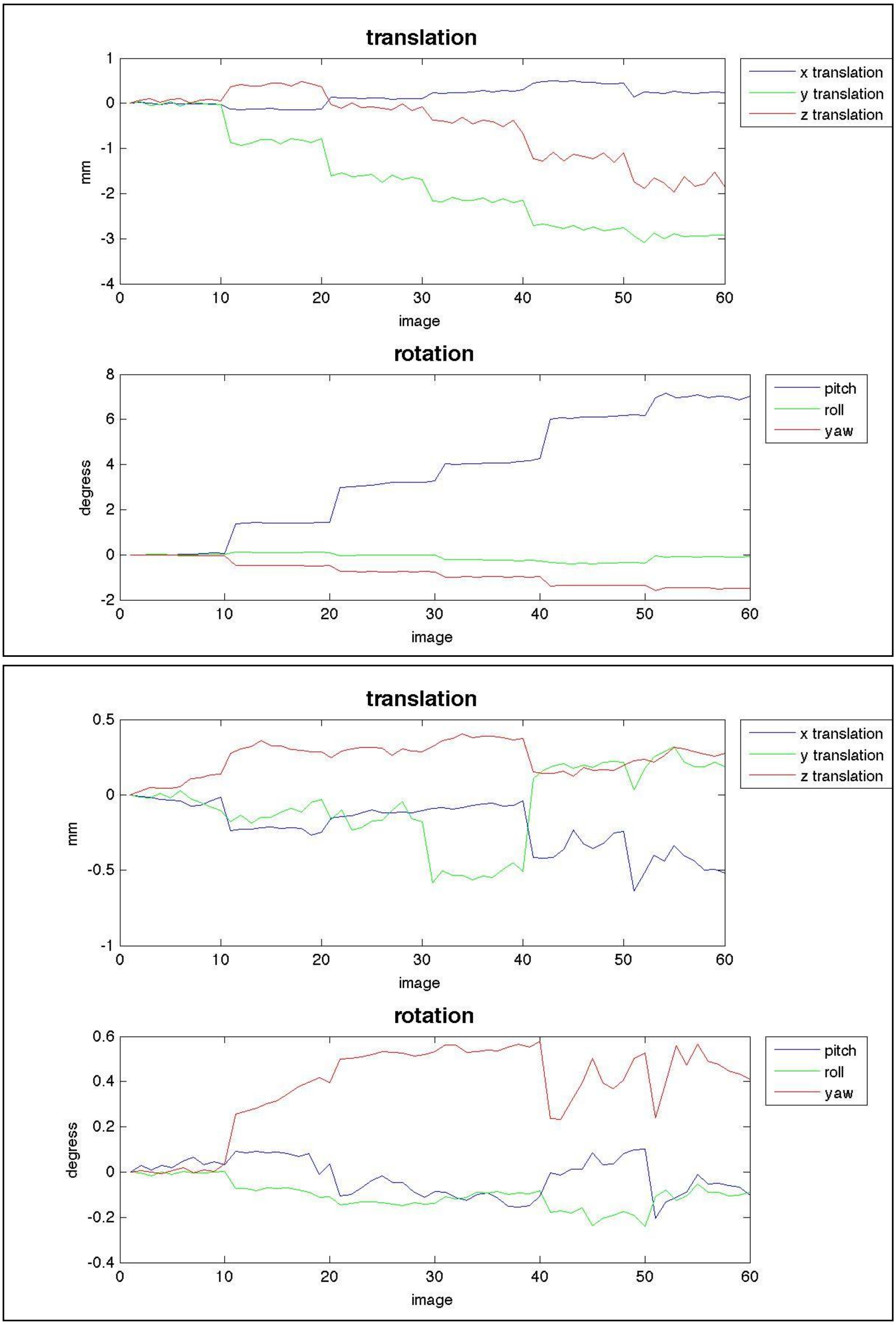

Figure S1. The 3 translational and 3 rotational components of the motion for the 2 example subjects in **Figure 3** of the paper when the transformation matrix is applied to the point at the center of the FOV. The subject with the largest maximum motion magnitude is shown at the top (translation followed by rotation) and the subject with the lowest maximum motion magnitude at the bottom. (The label “Image” on the x-axis is navigator number in the 6 sets of 10 navigators each.)
